# Health status measurement in COPD: the minimal clinically important difference of the clinical COPD questionnaire

**DOI:** 10.1186/1465-9921-7-62

**Published:** 2006-04-07

**Authors:** JWH Kocks, MG Tuinenga, SM Uil, JWK van den Berg, E Ståhl, T van der Molen

**Affiliations:** 1 Department of General Practice University Medical Center Groningen, The Netherlands; 2 Department of Pulmonary Diseases, Isala klinieken, Zwolle, The Netherlands; 3 AstraZeneca R&D, Lund, Sweden; 4 Primary Care Respiratory Medicine, University of Aberdeen, UK

## Abstract

**Background:**

Patient-reported outcomes (PRO) questionnaires are being increasingly used in COPD clinical studies. The challenge facing investigators is to determine what change is significant, ie what is the minimal clinically important difference (MCID). This study aimed to identify the MCID for the clinical COPD questionnaire (CCQ) in terms of patient referencing, criterion referencing, and by the standard error of measurement (SEM).

**Methods:**

Patients were ≥40 years of age, diagnosed with COPD, had a smoking history of >10 pack-years, and were participating in a randomized, controlled clinical trial comparing intravenous and oral prednisolone in patients admitted with an acute exacerbation of COPD. The CCQ was completed on Days 1–7 and 42. A Global Rating of Change (GRC) assessment was taken to establish the MCID by patient referencing. For criterion referencing, health events during a period of 1 year after Day 42 were included in this analysis.

**Results:**

210 patients were recruited, 168 completed the CCQ questionnaire on Day42. The MCID of the CCQ total score, as indicated by patient referencing in terms of the GRC, was 0.44. The MCID of the CCQ in terms of criterion referencing for the major outcomes was 0.39, and calculation of the SEM resulted in a value of 0.21.

**Conclusion:**

This investigation, which is the first to determine the MCID of a PRO questionnaire via more than one approach, indicates that the MCID of the CCQ total score is 0.4.

## Background

Chronic Obstructive Pulmonary Disease (COPD) is a major cause of morbidity and mortality in industrialized countries. COPD affects several organs and systems, and has a considerable impact on health status. Impaired exercise tolerance, exacerbations, fatigue, muscle weakness, depression and sleeping disorders are all features of the disease, and although spirometry is useful for assessing the effects of COPD on the lungs, it yields limited information relevant to health status or symptoms. Nevertheless, health status has become a central feature of studies in COPD in recent years because: (i) treatments for the condition are largely symptomatic, and (ii) European clinical trials are now required to incorporate a symptomatic measure[1,2]. The importance of the evaluation of health status in COPD has been demonstrated by two studies that show correlations between health status and other clinical outcomes. Poor scores on the St George's Respiratory Questionnaire (SGRQ), an instrument that measures disease specific health status, were associated with mortality, hospital readmission and increased healthcare resource consumption[3,4].

A number of questionnaires for the assessment of health-related quality of life and health status which cover a broader view of patients' well-being have been introduced into clinical practice since the late 1980s. These include COPD specific tools, such as the Chronic Respiratory Questionnaire (CRQ),[5] the SGRQ (which is for both asthma and COPD),[6] the generic instruments such as the Medical Outcomes Study Short-Form 36 (SF-36), [7] the Breathing Problems Questionnaire (BPQ)[8] and the Quality of Life for Respiratory Illness Questionnaire (QOL-RIQ)[9]. These instruments all capture valuable data, but have levels of complexity that make them difficult to use in the routine clinic setting. This has led to the need for a shorter and validated method to measure health status in order to assess clinical control in clinical trials as well as in daily clinical practice. The Clinical COPD Questionnaire (CCQ) [see Additional file: 1] has been developed to address this need[10].

One of the problems facing researchers using new assessments of patient-reported outcomes (PRO) questionnaires is the determination of what constitutes a change that can be considered significant [11]. This minimal clinically important difference (MCID) has been defined as 'the smallest difference in a score in the domain of interest which patients perceive as beneficial and which would mandate in the absence of troublesome side effects and excessive costs a change in the patient's management'[12]. The MCID can be determined by the judgment of the patient on the basis of a Global Rating of Change (GRC) questionnaire (patient referencing), by the clinician (clinician referencing – again with a global questionnaire), or by comparing scores on a health status instrument with a pre-specified health criterion (criterion referencing). These categories have been applied variously to other instruments such as the SGRQ and CRQ[4,6,12-14]. The aim of the present study was to identify the MCID for the CCQ in three different ways: patient referencing, criterion referencing, and by calculating the standard error of measurement (SEM), a method that seeks correlations between single standard error units and established MCID approximations [15,16].

## Patients and methods

### The CCQ

The CCQ is a 10-item, self-administered questionnaire that can be completed in less than 2 minutes. Items are divided into three domains: symptom, functional state and mental state; patients are required to respond to each item on a seven-point Likert scale where 0 = asymptomatic/no limitation and 6 = extremely symptomatic/total limitation. The final score is the mean of all ten items, and scores for the three domains can be calculated separately if required. Two versions are available: a 7-day version, which asks patients to recall their COPD status over the past week, and a 24-hour version, which is usually used as a diary. The CCQ has been validated and has shown strong discriminative properties, test-retest reliability and responsiveness[10].

### Patients

From June 2001 until May 2003, data were collected from 210 patients admitted to the Isala klinieken at Zwolle, The Netherlands with an acute exacerbation of COPD. These patients were participating in a randomized, controlled clinical trial designed to compare the effects of treatment with intravenous and oral prednisolone in patients with an acute exacerbation of COPD. Patients were at least 40 years of age and had COPD as indicated by the criteria of the American Thoracic Society[17]. All patients had a smoking history of more than 10 pack-years, and gave informed and written consent before enrolment.

Patients with a history of asthma were excluded, as were those with known hypersensitivity to prednisolone, chest X-ray not consistent with exacerbation of COPD, arterial PaCO_2 _above 9.3 kPa or acidosis (pH <7.26). Participation in another clinical trial in the four weeks preceding randomization, presence of severe co-morbidity, and inability to follow the investigator's instructions were also grounds for exclusion. Patients received either a 5-day course of continuous intravenous prednisolone (60 mg/24 hours diluted in 96 ml saline 0.9%) together with three-times daily one placebo tablet, or a 5-day course of three-times daily one tablet of 20 mg prednisolone with a continuous placebo infusion (100 ml saline 0.9%/24 hours). Active and placebo medication had a similar appearance. After 5 days all patients received oral prednisolone at a dosage of 30 mg once daily, which was subsequently reduced by 5 mg daily until 0 mg or a prior maintenance dosage was reached[18].

### Data collection

#### Patient referencing

The CCQ was completed on Days 1 to 7 and during an outpatient visit on Day 42. A GRC assessment was also taken on Days 2 and 3 to evaluate self-perceived changes in disease control since the first day of admission to hospital. Responses were scored from +7 (a very great deal better) to -7 (a very great deal worse); 0 indicated no change [22]. Scores of -3, -2, +2 and +3 were considered to represent minimal but nevertheless clinically important changes. To establish the MCID by patient referencing, the mean change in CCQ score from admission to Day 2 or 3 of the group with minimal change on the GRC questionnaire (-3, -2, +2 and +3) was calculated.

#### Criterion referencing

Health events were classified as major (hospital readmission for a pulmonary cause or death) or minor (worsening of COPD symptoms requiring treatment with an oral corticosteroid and/or antibiotics). Major health events only were included in the present analysis, with data pertaining to health events in all patients who completed the CCQ on Day 42 of the follow-up period. Data were obtained from general practitioners and hospital records.

#### SEM

SEMs were calculated using the following equation:[19]

SEM = σ_x _√1-*r*_xx_

Where (i) *r*_xx _= the reliability/intra class coefficient of the CCQ = 0.94;[10] and (ii) σ_x _= standard deviation of the total CCQ on Day 42 (baseline) = 0.87.

#### Follow-up

Patients were followed for 12 months after completion of the CCQ on Day 42 in order to collect data on health events that could be matched to CCQ responses. Electronic medical dossiers at the trial centre were checked and data were provided by general practitioners, with information requested including dosages and lengths of courses for oral corticosteroids and/or antibiotics, hospital admissions for COPD exacerbations, admission to nursing homes, and death.

### Statistical analysis

All analyses were performed with SPSS software version 12.0 (SPSS Inc., Chicago). A paired samples t-test was used to test the differences between CCQ total and domain scores on admission and on Days 2 and 3. The Wilcoxon signed ranks test was used for the mental state domain, since scores in this domain were skewed.

For criterion referencing, means and standard deviations of total, functional and symptom CCQ scores were calculated. Unpaired t-tests were used to compare differences between groups. For CCQ scores in the mental state domain, the non-parametric Mann-Whitney U test was used. P values less than 0.05 were considered to be statistically significant.

## Results

Of the 210 patients who were recruited to the clinical study on which this analysis is based, 168 completed the CCQ questionnaire on Day 42, 58 had global ratings of change for Day2, 59 on Day 3 and completed the CCQ on Day 1,2 and 3. Of the 168 patients who were followed up in the criterion referencing population, 24% were current smokers; the median smoking history across all these patients was 36.5 pack-years (range: 11 to 130 pack-years). Ages ranged from 43 to 84 years, with a median age of 71 years. Most patients were experiencing moderate (47.6%) or severe (33.3%) disease according to Global Obstructive Lung Disease (GOLD) criteria[20]. The 42 patients that could not be included in the criterion referencing study (14 withdrew their informed consent, 12 died before day 42, 9 were lost to follow-up, 5 had no CCQ data, 1 had no exacerbation and 1 reported side effects of study medication), were slightly older with a median of 74 years, but were similar in respect to percentage predicted forced expiratory volume in 1 second (FEV_1_), current smoking and number of pack-years.

As might be expected as a result of study intervention, the FEV_1 _increased significantly in the criterion referencing population from 37.7% to 43.2% (means, p = 0.000) between hospital admission (Day 1) and Day 42.

### Patient referencing

Tables 1 and 2 show mean CCQ changes between Days 2 and 3, respectively, grouped according to response on the GRC scale. Twenty-one patients responded with no change and 3 reported worsening on Day 2. On Day 3, 1 patient reported worsening whereas 10 patients reported no change. The first category, which shows changes of +1 (no discernable or only very slight improvement), included only very small numbers of patients on both Days 2 and 3 and is below the threshold for clinical change specified in the protocol. No significant change in CCQ scores for any domain was seen for this category. However, at the threshold for clinically relevant change (score change of +2 or +3), some significant improvements in CCQ scores became apparent: on Day 2, CCQ changes of 0.70 for the symptom domain and 1.0 for the mental domain fell outside the respective 95% confidence intervals and attained statistical significance. A trend towards significant change for the total CCQ score on Day 2 (0.40; p = 0.098) translated into statistically significant improvement on Day 3 (0.44; p = 0.008) that was associated with a GRC improvement of +2 or +3. Statistical significance was maintained on Day 3 for the symptom domain, but was lost for the mental domain. Furthermore, the number of patients available for CCQ scoring in the GRC +2 or +3 category increased from 15 on Day 2 to 20 on Day 3. These observations therefore suggest that the MCID of the CCQ total score, as indicated by patient referencing in terms of the GRC, is 0.44.

**Table 1:** Minimal Clinically Important Difference (MCID) for the Clinical COPD Questionnaire (CCQ) by patient referencing. Differences between CCQ scores for Days 1 and 2 grouped according to Global Rating of Change (GRC) as scored by patients on a scale of -7 to 7. Note that paired-sample t-tests were used for total CCQ scores and for symptom and functional domains; Wilcoxon signed rank test was used for the mental domain.

CCQ score category	Score difference: Day 1 minus Day 2		
	
	Mean ± SD	95% confidence interval	p value
**GRC +1 (n = 3)**			

Total	0.30 ± 0.30	-0.45, 1.05	0.225
Symptoms	-0.25 ± 1.64	-4.32, 3.82	0.816
Function	1.17 ± 0.80	-0.83, 3.16	0.128
Mental	-0.5§		0.157

**GRC +2 to +3 (n = 15)**			

Total	0.40 ± 0.90	-0.09, 0.91	0.098
Symptoms	0.70 ± 1.09	0.98, 1.30	0.026*
Function	0.17 ± 1.37	-0.93, 0.59	0.645
Mental	1.0§		0.007*

**GRC +4 to +5 (n = 14)**			

Total	1.31 ± 1.09	0.69, 1.94	0.001*
Symptoms	1.13 ± 1.52	0.25, 2.0	0.016*
Function	1.48 ± 1.25	0.76, 2.20	0.001*
Mental	0.5§		0.018*

**GRC +6 to +7 (n = 2)**			

Total	1.95 ± 0.07	1.31, 2.59	0.016*
Symptoms	2.75 ± 1.77	-13.13, 18.63	0.272
Function	1.50 ± 1.06	-8.03, 11.03	0.295
Mental	1.25§		0.317

* Statistically significant (2-tailed): p < 0.05. § Difference in median scores

**COPD **= chronic obstructive pulmonary disease; **SD **= standard deviation.

**Table 2:** Minimal Clinically Important Difference (MCID) for the Clinical COPD Questionnaire (CCQ) by patient referencing. Differences between CCQ scores for Days 1 and 3 grouped according to Global Rating of Change (GRC) as scored by patients on a scale of -7 to 7. Note that paired-sample t-tests were used for total CCQ scores and for symptom and functional domains; Wilcoxon signed rank test was used for the mental domain.

CCQ score category	Score difference: Day 1 minus Day 3		
	
	Mean ± SD	95% confidence interval	p value
**GRC +1 (n = 4)**			

Total	0.05 ± 0.49	-0.73, 0.83	0.852
Symptoms	0 ± 1.02	-1.62, 1.62	1.0
Function	0 ± 1.02	-1.62, 1.62	1.0
Mental	0.25§		0.414

**GRC +2 to +3 (n = 20)**			

Total	0.44 ± 0.66	0.13, 0.75	0.008*
Symptoms	0.74 ± 0.93	0.30, 1.17	0.002*
Function	0.26 ± 0.995	-0.20, 0.72	0.253
Mental	0.25§		0.398

**GRC +4 to +5 (n = 20)**			

Total	1.36 ± 1.07	0.86, 1.86	<0.001*
Symptoms	1.46 ± 0.99	1.0, 1.92	<0.001*
Function	1.31 ± 1.65	0.54, 2.08	0.002*
Mental	1.75§		0.023*

**GRC +6 to +7 (n = 4)**			

Total	1.95 ± 0.87	0.56, 3.34	0.021*
Symptoms	2.37 ± 1.13	0.58, 4.17	0.024*
Function	2.31 ± 0.90	0.88, 3.74	0.014*
Mental	-0.25§		0.655

* Statistically significant (2-tailed): p < 0.05. § Difference in median scores

**COPD **= chronic obstructive pulmonary disease; **SD **= standard deviation.

As might be expected, significant improvements in all CCQ domains were seen in the GRC category of +4 to +5 (Table 1 and Table 2). These GRC scores represent higher levels of patient-perceived clinical improvement that are reflected by significant improvements in CCQ scoring (CCQ changes ranged from 1.25 to 1.46 across domains on Day 3), but are too great to be considered as minimally clinically relevant. Too few patients were represented in the maximal GRC change category (+6 to +7) on Days 2 and 3 for CCQ results to be trustworthy, but there was an overall trend towards further increases in CCQ scores.

### Criterion referencing

Differences in mean CCQ scores on Day 42 between patients who experienced major health events (death, rehospitalization and death and/or rehospitalization) during the subsequent 12 months are presented in Table 3. There were no significant differences that could be related to clinical outcomes in the mental domain of the CCQ, but changes of interest were seen for functioning and symptoms, and for total CCQ scores.

**Table 3:** Minimal Clinically Important Difference (MCID) for the Clinical COPD Questionnaire (CCQ) by criterion referencing. Differences between baseline (Day 42) CCQ scores are grouped according to major health events during 12-month follow-up. Unpaired-sample t-tests were used for total CCQ scores and for symptom and functional domains, with equal variances assumed; the Mann-Whitney U test was used for the mental domain.

CCQ score category	Score difference		
	
	Mean	95% confidence interval	p value
**Death (n = 25) or survival (n = 143)**			

Total	-0.80	-1.23, -0.37	<0.001*
Symptoms	-0.62	-1.13, -0.12	0.015*
Function	-1.32	-1.90, -0.74	<0.001*
Mental	0.5§		0.211

**Rehospitalization (n = 56) or not (n = 112)**			

Total	-0.18	-0.52, 0.16	0.290
Symptoms	-0.07	-0.46–0.31	0.706
Function	-0.47	-0.93, -0.007	0.047*
Mental	0§		0.505

**Death/rehospitalization (n = 70) versus survival/no rehospitalization (n = 98)**			

Total	-0.39	-0.71, -0.07	0.017*
Symptoms	-0.27	-0.63, 0.10	0.153
Function	-0.77	-1.19, -0.34	0.001*
Mental	0§		0.987

* Statistically significant (2-tailed): p < 0.05. § Difference in median scores

**COPD **= chronic obstructive pulmonary disease.

Day 42 total CCQ score difference was -0.8 between patients who died and those who survived over the next 12 months (p < 0.001). CCQ differences for rehospitalization were not as marked, however, with borderline significance being noted for the difference of -0.47 in the function domain (p = 0.047) only. For the combined major outcome of death and/or rehospitalization, a difference -0.39 for the total CCQ score, attained statistical significance (Table 3). Thus, the MCID by inspection for the CCQ in terms of criterion referencing for the major outcomes covered in this analysis is 0.39.

### SEM

Calculation of the SEM using the described method resulted in a SEM of 0.21.

## Discussion

The methods used in the present analysis to determine the MCID for the CCQ yielded similar findings with patient and criterion referencing (0.44 and 0.39 units respectively). However the SEM was much lower (0.21). In light of these observations, we suggest that the MCID of the CCQ instrument is approximately 0.4 points. Thus, a change in score of 0.4 or more from baseline indicates the smallest change indicated by the CCQ in health status that can be considered to be clinically significant.

The first method used, patient referencing, was based on CCQ changes linked to a prespecified global rating of change of +2 to +3 points over the first 3 days of treatment. In both this group and that with the next level of improvement (GRC change of +4 to +5), sufficient numbers of patients were available for clear patterns of change in the CCQ to be evident. The very small numbers of patients and consequent inconclusive results in the groups showing least (GRC change +1) and most (GRC change +6 to +7) clinical improvement was of little importance in the setting of the present analysis, as the change in health status of these patients was either too small or too large to be of interest.

Patient referencing has been used extensively by other investigators calculating MCIDs of PRO instruments, and our results are in broad agreement with these other findings. Furthermore, although this approach has not been formally validated, there is ample evidence that the global assessments used correlate well with PRO questionnaires.

Jaeschke and colleagues[12] performed an analysis in 55 patients with COPD who had participated in two clinical trials and in 20 patients with heart failure. Changes in CRQ[5] and Chronic Heart Failure Questionnaire (CHQ)[21] scores were compared with retrospective global estimates of change by the patients themselves on a 15-point transition scale similar to our GRC (seven categories of improvement, seven of deterioration and one of no change). The authors set the threshold for clinical significance on this scale as 'almost the same, hardly any better (or worse)', 'a little better (or worse)' or 'somewhat better (or worse)', the last two of which approximate to the change of 2 to 3 on the scale used here. Although there was considerable variation between patients in MCID estimates, mean changes corresponding to the predefined threshold were 0.43 for dyspnea, 0.64 for fatigue, and 0.49 for emotional function. Jaeschke and colleagues[12] concluded that the mean change in score per question that corresponded to the MCID was consistently around 0.5.

Juniper et al[22] adopted a similar approach to determine an MCID for the Asthma Quality of Life Questionnaire (AQLQ), except that their threshold for minimally significant change was more similar to ours than that adopted by Jaeschke et al:[12] AQLQ scores that corresponded to 'a little better (or worse)' and 'somewhat better (or worse)' were used. In this analysis, each of 39 patients attending an asthma clinic was followed for 8 weeks. For overall asthma-specific quality of life and for all individual domains (activities, emotions and symptoms), the MCID per item was close to 0.5 (0.42 to 0.58). Differences of approximately 1.0 corresponded to moderate change, and large changes were accompanied by score changes of around 1.5.

It is worth noting at this point that more noticeable global changes as shown by the GRC were accompanied in our analysis by larger CCQ changes. By Day 3, a GRC of +4 to +5 was associated with mean increases in CCQ scores of 1.25 to 1.46 for the separate domains, and an increase in total mean score of 1.36. These changes were consistent across domains and were all statistically significant.

Further data in patients with asthma are available from a 1-year study in which 719 adults received nedocromil sodium or placebo and were assessed with the SGRQ[23]. Differences in scores from baseline to 12 months were compared with patients' own retrospective estimates of treatment efficacy, and there was a rank order correlation between change in health-related quality of life and overall judgement of treatment efficacy. Patients who judged treatment to be 'slightly effective' showed a mean 4.0-unit change on the SGRQ[24]. In another study,[25] 87 patients who judged treatment with salmeterol to be 'satisfactory' showed a mean change in SGRQ of 2.0 points over 16 weeks. This term, however, was deemed ambiguous[13]. The lowest response category compatible with efficacy, 'effective', corresponded with a mean SGRQ change of 4.3 units in 109 patients.

Although it is not possible to compare these authors' results with those reported here because of the different PRO questionnaires and health status scales examined, it is clear that all these investigators were readily able to identify MCIDs by patient referencing methods. Furthermore, patterns of findings across the different studies are remarkably consistent, and show not only the smallest discernible changes, but also consistent increases in health status scores in parallel with patients' own perceptions of greater clinical improvement.

The criterion referencing approach compares health status scores to a specified health-related variable on the understanding that PRO questionnaire scores should be worse in patients who have major health events than in those who do not. Upon examination of CCQ scores categorized according to the major health outcomes of death, rehospitalization, and death and/or rehospitalization, we found the smallest statistically significant score change associated with one of these outcomes to be 0.39. Score changes that exceeded this value were found to be consistently significant, while lower scores failed to attain significance.

It should also be noted that MCIDs determined by this method might be expected to have predictive value, as the CCQ score differences were noted at baseline point of study Day 42 and corresponded to subsequent health outcomes reported 1 year later. Thus, it can be concluded that when a difference in CCQ score between two patients with COPD exceeds 0.39 points, the patient with the higher score has an increased risk of dying and/or being readmitted to hospital during the course of the following year. Overall, the smallest CCQ differences were found to be those between patients who were readmitted to hospital and those who were not, whereas the score differences between patients who died and those who survived were the largest. This predictive value concurs with results of other studies, such as Domingo-Salvany et al.[3] who reported a link between reduced duration of survival for male patients with COPD and poor health-related quality of life. In addition, Osman and colleagues[4] found poor SGRQ scores to be associated with increased risk of hospital readmission for COPD.

The similarity between MCIDs determined by patient and criterion referencing for the CCQ as noted in the present analysis has been apparent in research into other health-related quality of life scales. SGRQ scores at baseline differed by 4.8 units between patients who were admitted to hospital or died and those who experienced neither of these outcomes in the year following discharge from hospital for an acute exacerbation of asthma in a study in 238 individuals[4]. Similarly, in a study in patients with COPD, SGRQ scores were related to Medical Research Council dyspnea gradings[26]. In 32 patients with a grading of 5 (housebound), SGRQ scores were 3.9 units worse than in patients who had major impairment but who were not housebound (grade 4).

The SEM has not been used in many studies for establishing the MCID of PRO questionnaires. For the CRQ, one-SEM appears to be closely related to the MCID of the CRQ[15]. In this study the SEM was found to be 0.21, which is lower than the other two methods used for establishing the MCID of the CCQ. This might be because of the high reliability/intra-class coefficient. Some researches take a more conservative approach to the assessment of the MCID using the SEM. They use the 1.96 SEM, which represents a 95% confidence interval[19]. Using this conservative measure, the MCID is 0.41, a similar result to that produced by the two other methods.

Thus, the present investigation, which is the first to determine the MCID of a PRO questionnaire via more than one approach, indicates that the MCID of the CCQ total score is 0.4. Our findings also demonstrate the predictive value of such differences in terms of longer term major health outcomes in patients with COPD.

## Competing interests

JK and MT received an unrestricted grant by AstraZeneca Sweden. ES is employed by AstraZeneca Sweden, TvdM has received research grants from Altana Pharma, AstraZeneca, Boehringer Ingelheim and GlaxoSmithKline, and speakers fees from AstraZeneca, AltanaPharma and GlaxoSmithKline.

## Authors' contributions

JK: analysis, interpretation and writing manuscript; MT: design, data collection, interpretation, revising manuscript; SU: design, data collection, revising manuscript; JB: data collection, revising manuscript; ES: design, interpretation, revising manuscript; TvdM: design, interpretation, drafting and revising manuscript. All authors read and approved the final manuscript.

## Supplementary Material

Additional file 1 Click here for file
