# Supplementary material for: Health status measurement in COPD: the minimal clinically important difference of the clinical COPD questionnaire
Source: Respir Res. 2006 Apr 7;7(1):62. doi: 10.1186/1465-9921-7-62 (PMC1508149; doi:10.1186/1465-9921-7-62)
Supplement: Additional file 1 [file 1465-9921-7-62-S1.PDF]

CLINICAL COPD QUESTIONNAIRE

© 1999

Information:
Prof. T. van der Molen, MD PhD
Dept. Of General Practice
University Medical Center Groningen
Postbus 196
9700 AD Groningen
The Netherlands
Fax: +31 503632964
E-mail: t.van.der.molen@med.umcg.nl
Internet: www.ccq.nl

© The CCQ is copyrighted. It may not be altered, sold (paper or electronic), translated or adapted for another medium without the permission of T. van der Molen, Dept. Of General Practice, University Medical Center Groningen, Postbus 196, 9700 AD Groningen, The Netherlands.

Patient number:_________
Date:_________________

CLINICAL COPD QUESTIONNAIRE

Please **circle** the number of the response that best describes how you have been feeling during the **past week**. (Only **one** response for each question).

On average, **during the past week**, how often did you feel:	never	hardly ever	a few times	several times	many times	a great many times	almost all the time
1. Short of breath **at rest**?	0	1	2	3	4	5	6
2. Short of breath **doing physical activities**?	0	1	2	3	4	5	6
3. **Concerned** about getting a cold or your breathing getting worse?	0	1	2	3	4	5	6
4. **Depressed (down)** because of your breathing problems?	0	1	2	3	4	5	6
In general, **during the past week**, how much of the time:							
5. Did you **cough**?	0	1	2	3	4	5	6
6. Did you **produce phlegm**?	0	1	2	3	4	5	6
On average, **during the past week**, how limited were you in these activities **because of your breathing problems**:	not limited at all	very slightly limited	slightly limited	moderately limited	very limited	extremely limited	totally limited /or unable to do
7. **Strenuous physical activities** (such as climbing stairs, hurrying, doing sports)?	0	1	2	3	4	5	6
8. **Moderate physical activities** (such as walking, housework, carrying things)?	0	1	2	3	4	5	6
9. **Daily activities at home** (such as dressing, washing yourself)?	0	1	2	3	4	5	6
10. **Social activities** (such as talking, being with children, visiting friends/ relatives)?	0	1	2	3	4	5	6

© The CCQ is copyrighted. It may not be altered, sold (paper or electronic), translated or adapted for another medium without the permission of T. van der Molen, Dept. Of General Practice, University Medical Center Groningen, Postbus 196, 9700 AD Groningen, The Netherlands.
